# Supplementary material for: Depth dependent stress revealed by aftershocks
Source: Nat Commun. 2017 Nov 6;8:1317. doi: 10.1038/s41467-017-01446-y (PMC5673070; doi:10.1038/s41467-017-01446-y)
Supplement: Supplementary file 1 — Supplementary Information [file 41467_2017_1446_MOESM1_ESM.pdf]

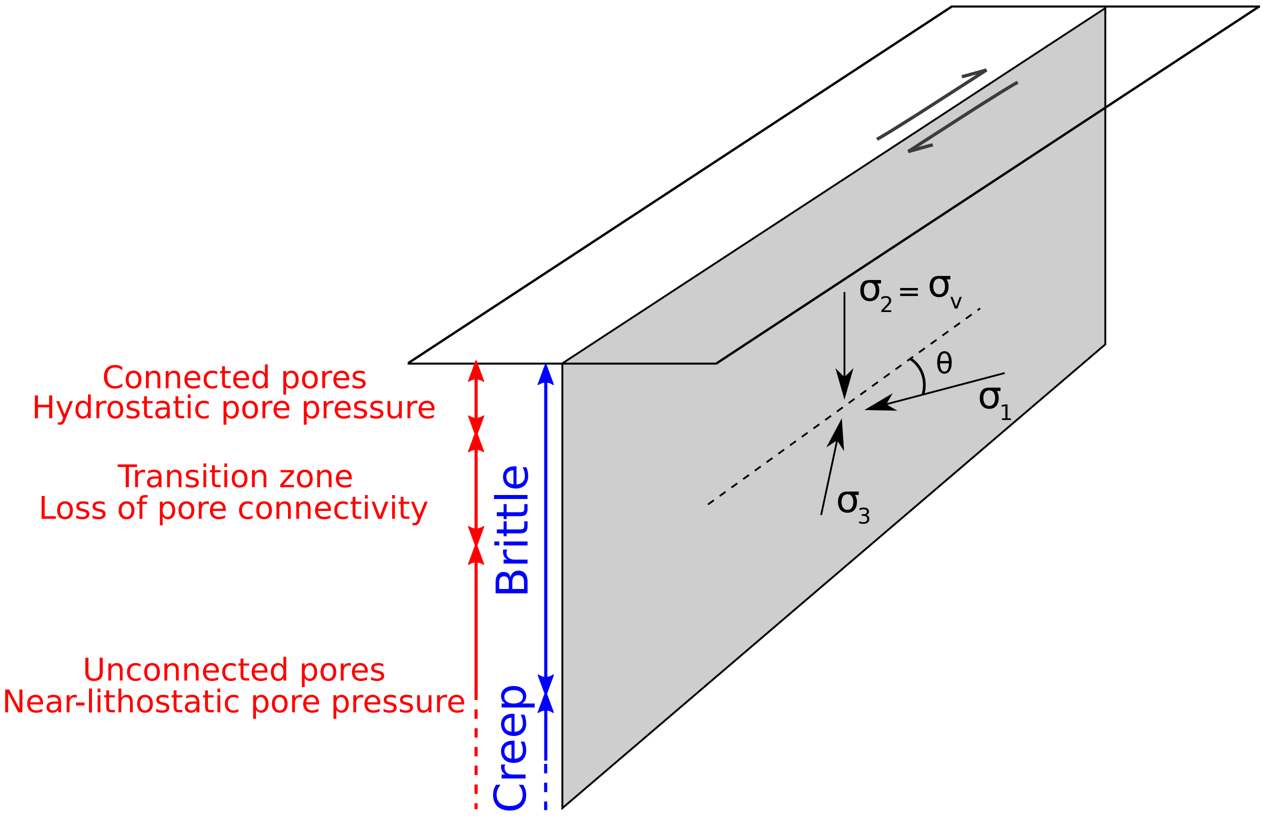


**Supplementary Figure 1:** Conceptual scheme for strike-slip faulting along the San Andreas fault system. Black arrows show the principal shear stresses on a vertical strike-slip fault according to the Anderson's theory of faulting. For the model derived from aftershock data, blue and red arrows show transitions in deformation regime and pore-fluid pressure with respect to depth, respectively.

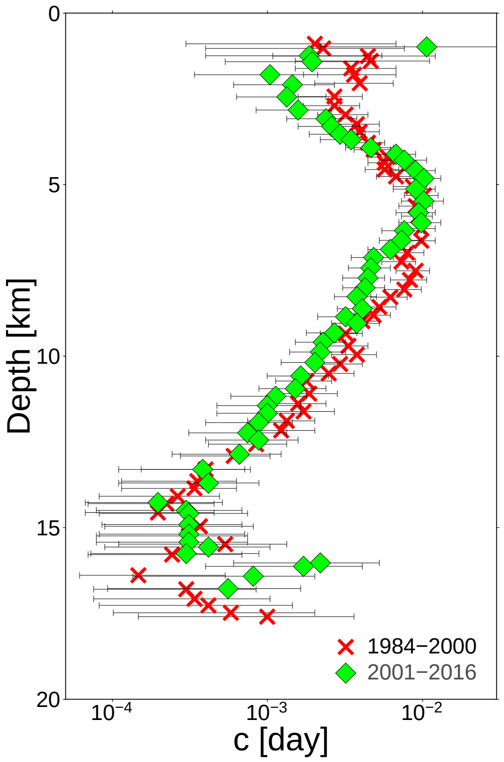


**Supplementary Figure 2:** Vertical $c$-value profiles for two time periods considering together all the events occurring along major sub-vertical strike-slip faults in California: 1984-2000 (red crosses); 2001-2016 (green diamonds). Error bars show the 95% Bayesian credibility regions for the $c$-value.

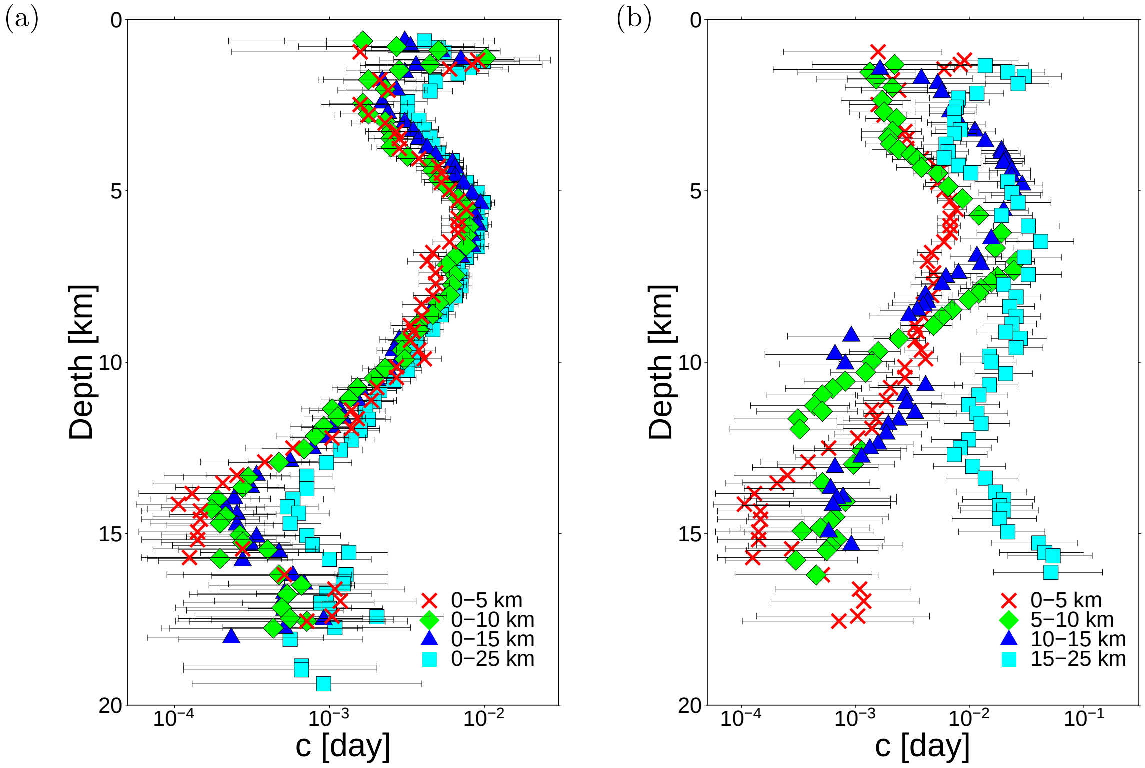


**Supplementary Figure 3:** Variations of the vertical $c$-value profile across the fault zone, considering together all the events occurring along major sub-vertical strike-slip faults in California: (a) Vertical $c$-value profiles for an increasing width for data selection from both sides of the faults; (b) Vertical $c$-value profiles in sidebands on both sides of the faults. Error bars show the 95% Bayesian credibility regions for the $c$-value.

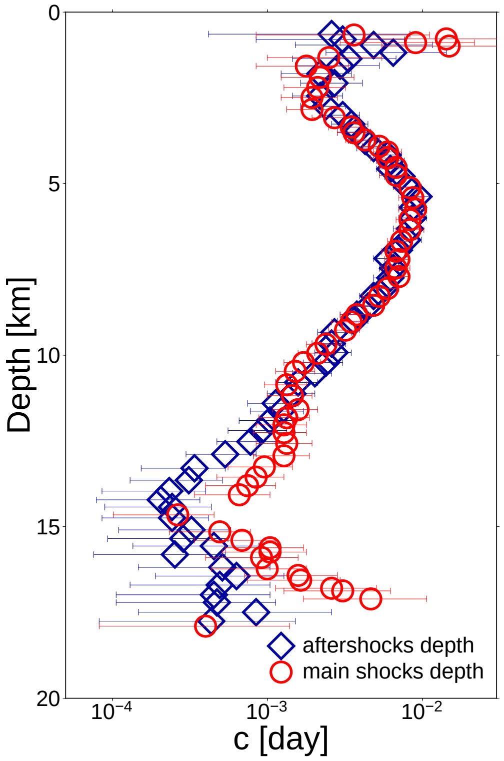


**Supplementary Figure 4:** Evolution of the $c$-value with respect to the average depth of aftershocks (blue diamonds) and mainshocks (red circles) considering together all the events occurring along major sub-vertical strike-slip faults in California. Error bars show the 95% Bayesian credibility regions for the $c$-value.

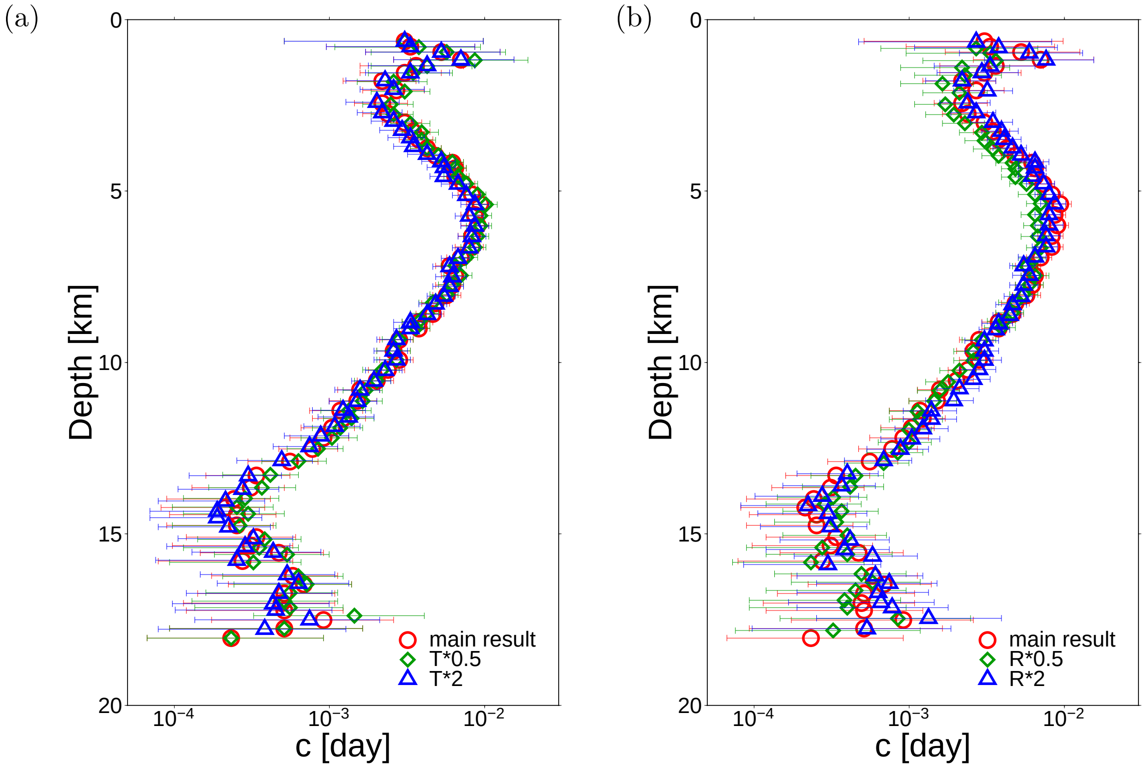


**Supplementary Figure 5:** Vertical $c$-value profiles using different parameterizations of the declustering procedure considering together all the events occurring along major sub-vertical strike-slip faults in California: (a) Influence of the spatial window size; (b) Influence of the temporal window duration. Error bars show the 95% Bayesian credibility regions for the $c$-value.

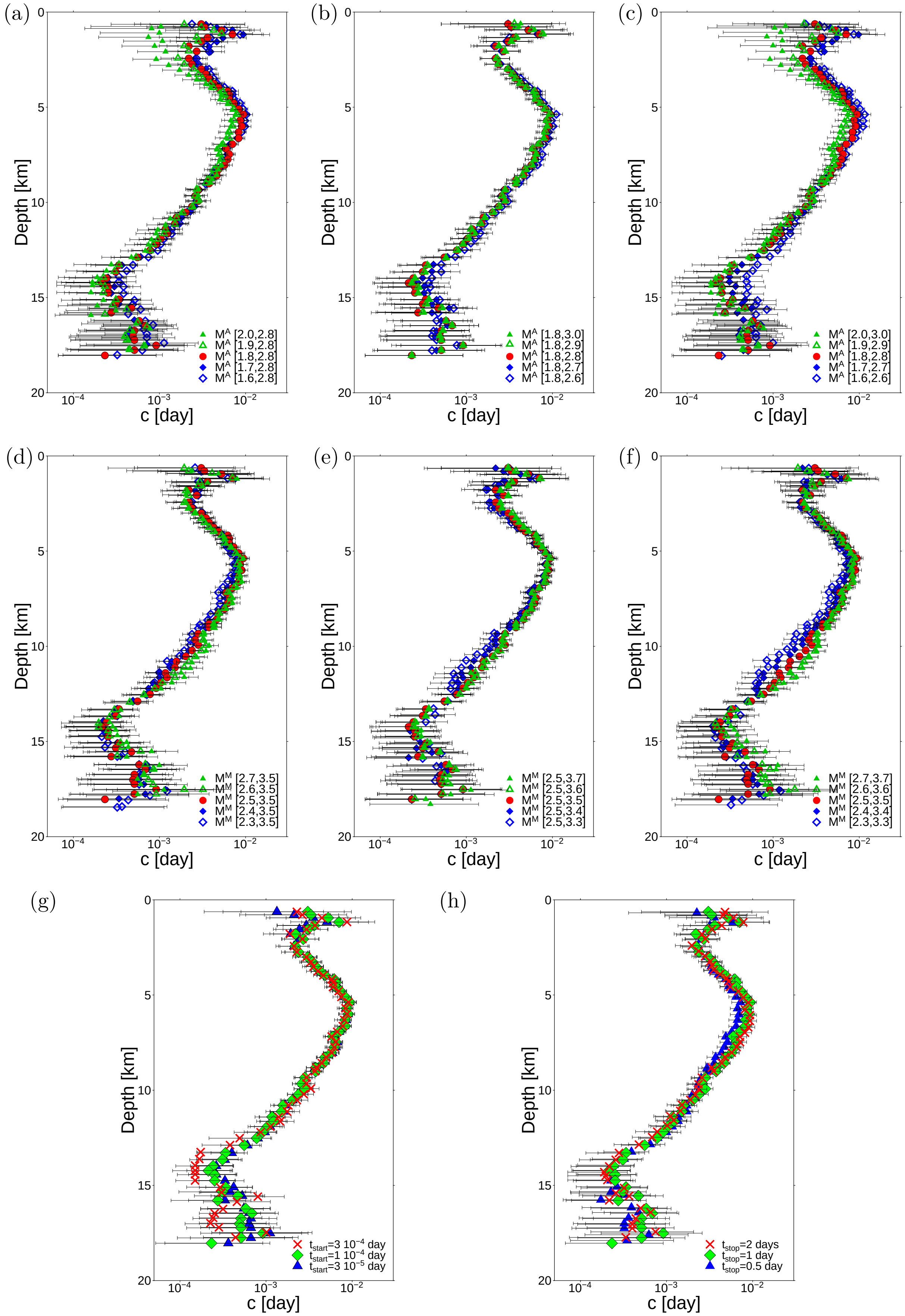


**Supplementary Figure 6:** Vertical $c$-value profiles using different magnitude ranges for aftershocks (a-c), mainshocks (d-f) and different time intervals (g-h). Here we consider together all the events occurring along major subvertical strike-slip faults in California. Plots show the sensibility to (a) $M_A^{min}$ the minimum aftershock magnitude, (b) $M_A^{max}$ the maximum aftershock magnitude, (c) $[M_A^{min}, M_A^{max}]$ the aftershock magnitude range, (d) $M_M^{min}$ the minimum mainshock magnitude, (e) $M_M^{max}$ the maximum mainshock magnitude, (f) $[M_M^{min}, M_M^{max}]$ the mainshock magnitude range, (g) $t_{start}$ and (h) $t_{stop}$ the lower and upper bounds of the time interval, respectively. Error bars show the 95% Bayesian credibility regions for the $c$-value.

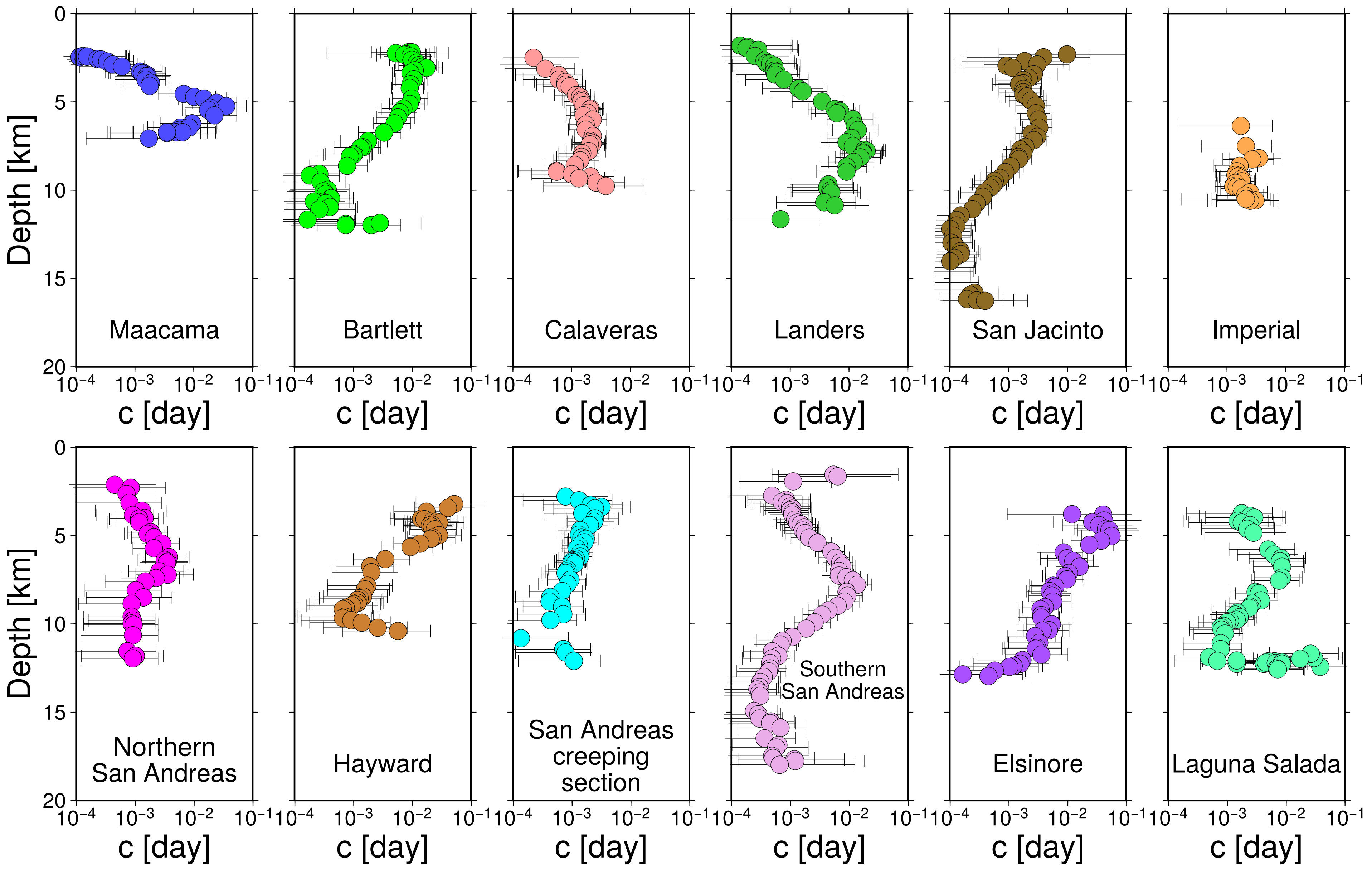


**Supplementary Figure 7:** Vertical $c$-value profile along 12 sub-vertical strike-slip fault segments in California using a declustering method based on nearest-neighbour distance in time-space-magnitude domains. Stacked aftershock sequences are only composed of first generation offspring events using $\{\nu_0, b, d_f\} = \{10^{-5}, 1, 1.6\}$ in Eq. 9 of the 'Methods' section. These $c$-value profiles can be directly compared to those shown in Fig. 2 of the main manuscript. Error bars show the 95% Bayesian credibility regions for the $c$-value.

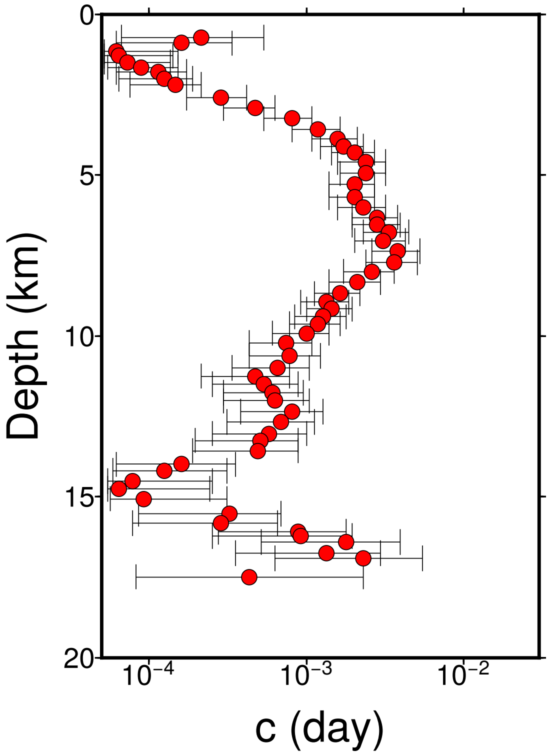


**Supplementary Figure 8:** Vertical $c$-value profile using a declustering method based on nearest-neighbour distance in time-space-magnitude domains. Stacked aftershock sequences are only composed of first generation offspring events using $\{\nu_0, b, d_f\} = \{10^{-5}, 1, 1.6\}$ in Eq. 9 of the 'Methods' section. Considering together all the major sub-vertical strike-slip faults in California shown in Fig. 2 of the main manuscript, this vertical profile can be directly compared to the profile shown in Fig. 4 of the main manuscript. Error bars show the 95% Bayesian credibility regions for the $c$-value.

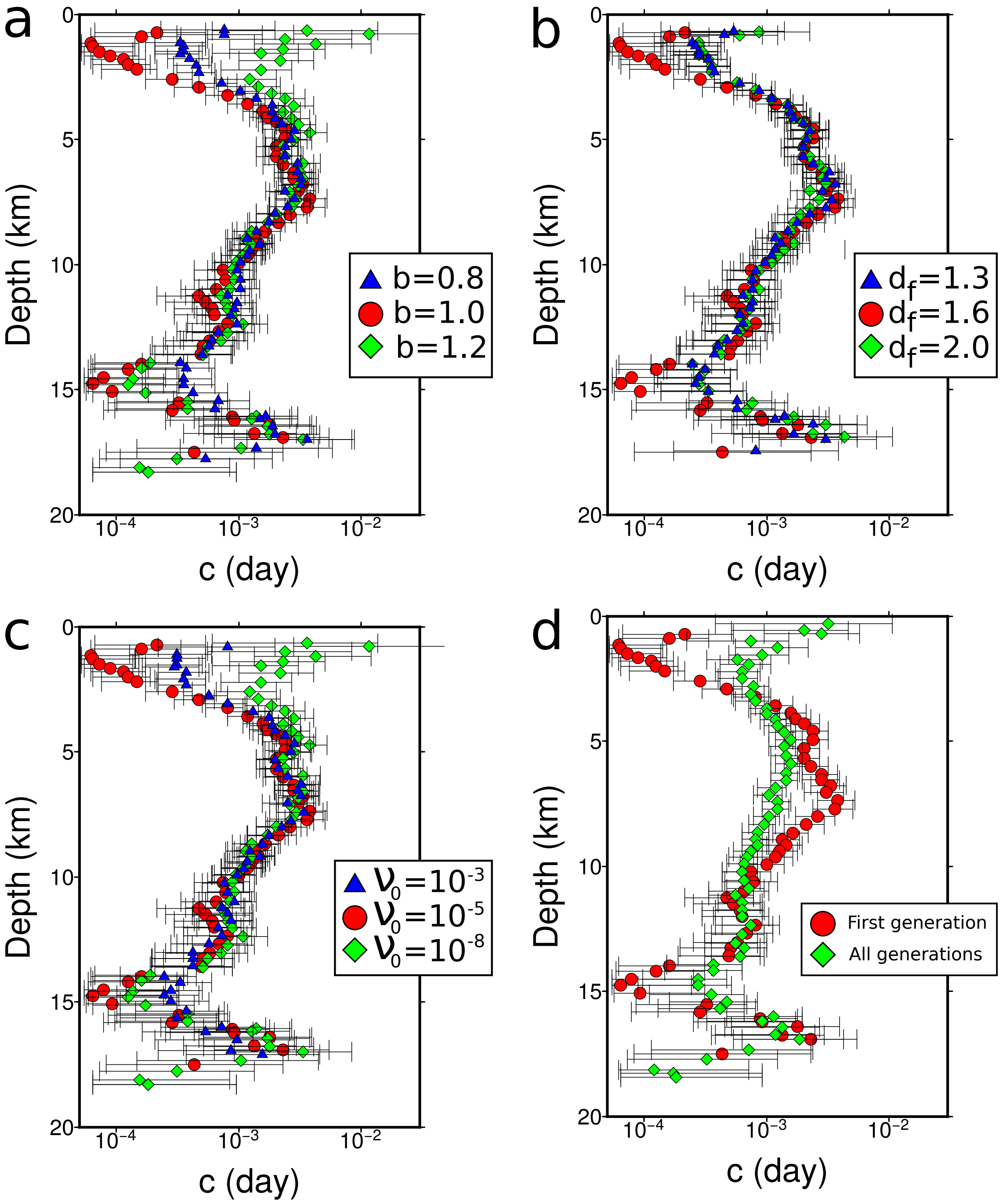


**Supplementary Figure 9:** Sensibility of the vertical $c$-value profile on the parameterization of the declustering method based on nearest-neighbour distance in time-space-magnitude domains: (a) slope $b$ of the earthquake-size distribution; (b) fractal dimension $d_f$ of the epicenters; (c) threshold $\nu_0$ for the proximity function; (d) different generations offspring events. The red dots are always for first generation offspring events using $\{\nu_0, b, d_f\} = \{10^{-5}, 1, 1.6\}$ in Eq. 9 of the 'Methods' section.

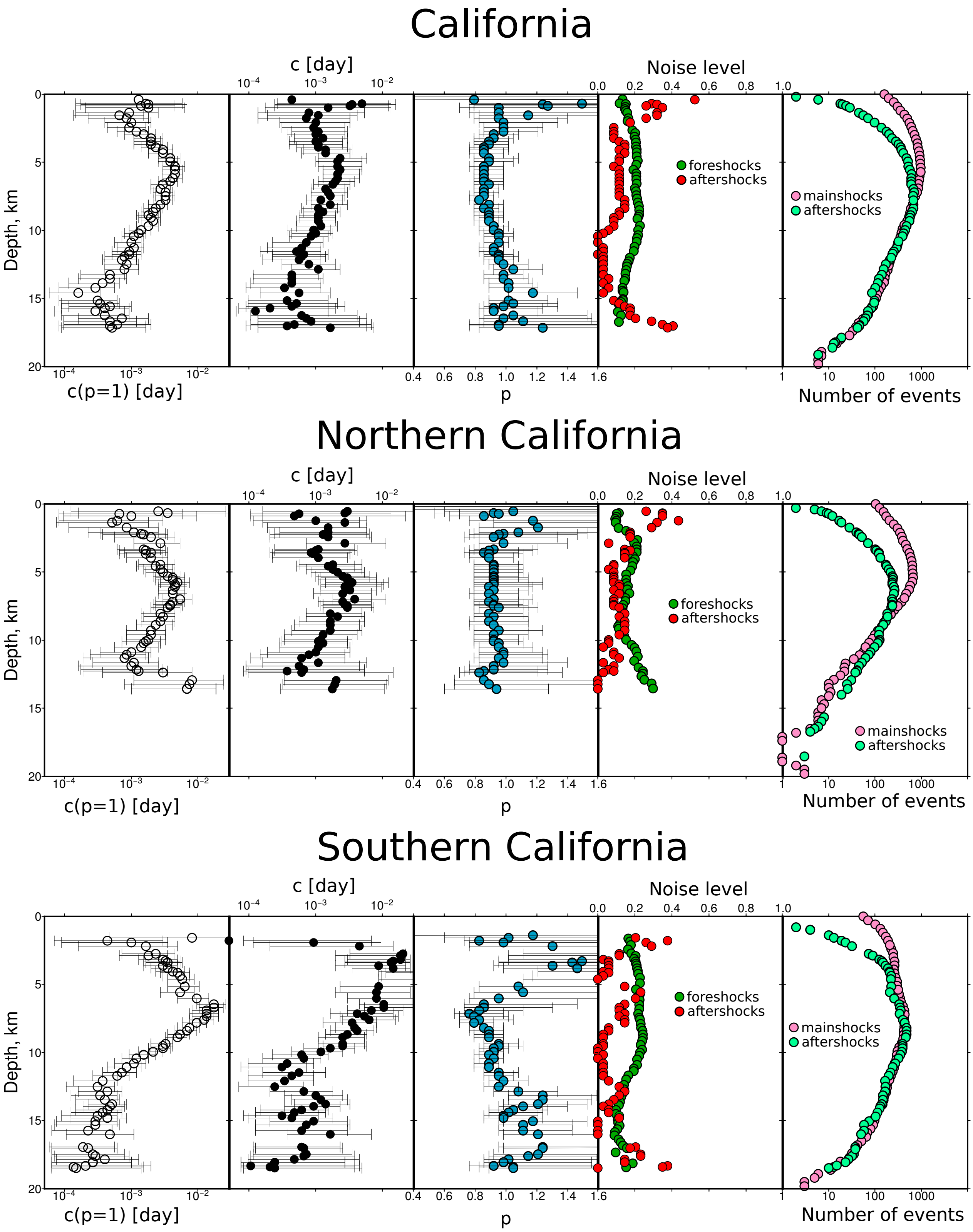


**Supplementary Figure 10:** Influence of depth on the aftershock decay rate in zones of strike-slip faulting for the entire California (top) and separately for northern (middle) and southern California (bottom). Left figures show the most probable $c$-values for $p = 1$ considering background activity in the Bayesian procedure (white dots). Using the same procedure without fixing $p$, central figures show the most probable $c$-value (black dots), $p$-value (blue dots) and the noise levels. The noise level for aftershocks (red dots) is the proportion of aftershocks associated with the background activity. The noise level for foreshocks (dark green dots) is the ratio between the number of events one day before and after the mainshocks selecting these so-called foreshocks as aftershocks. Right figures show the number of selected mainshocks (pink dots) and aftershocks (light green dots).

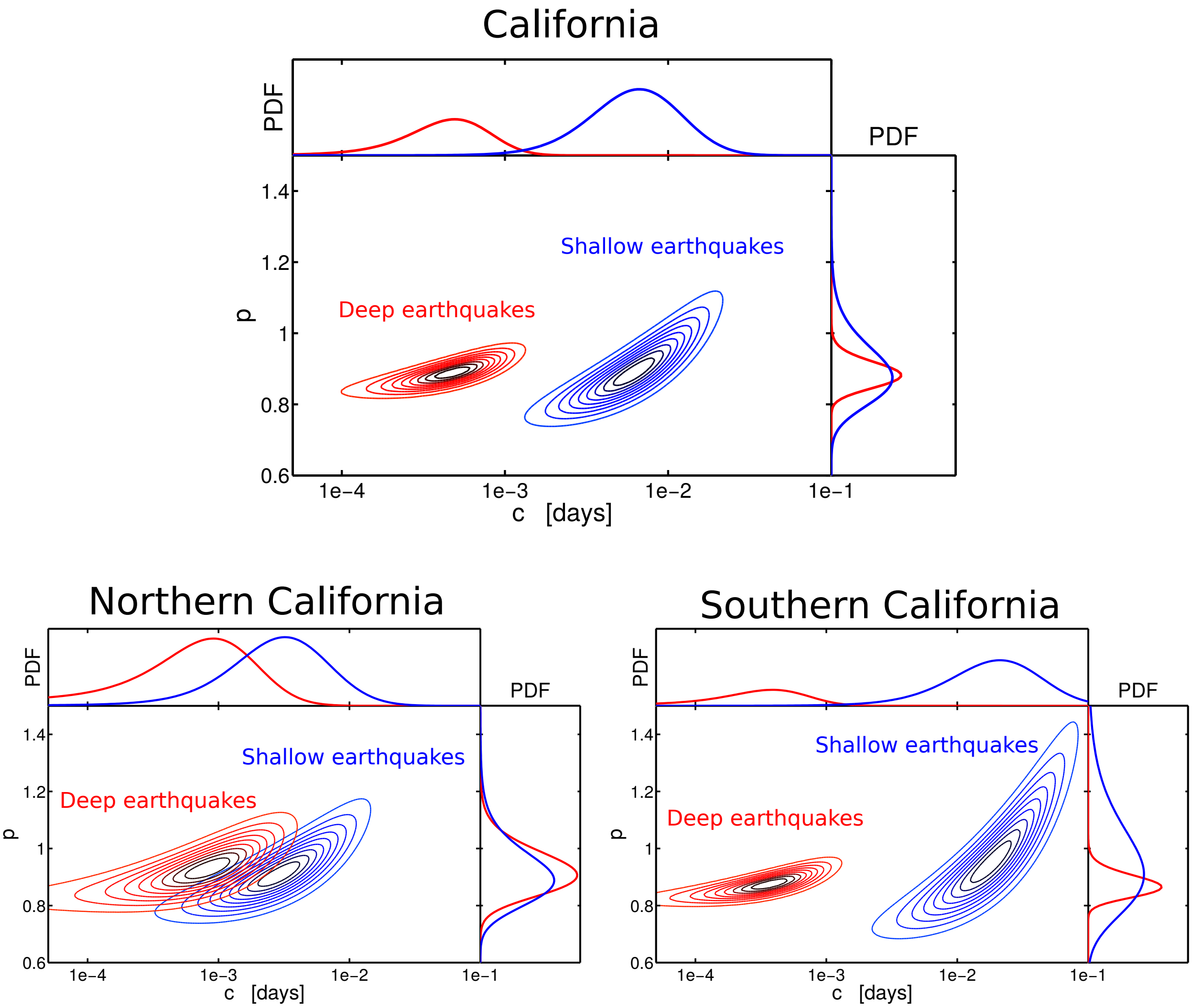


**Supplementary Figure 11:** Influence of depth on the aftershock decay rate in zones of strike-slip faulting for the entire California (top) and separately for northern (bottom right) and southern California (bottom left). Bayesian posterior densities of $\{c, p\}$ in two depth intervals of 4-6 km (blue, 557 events) and 9-13 km (red, 556 events) considering a single stack of aftershocks for all the major strike-slip faults. Contours are the deciles of each distribution. Curves show the probability distribution functions of the marginal posterior distributions of $p$ (left) and $c$ (top).

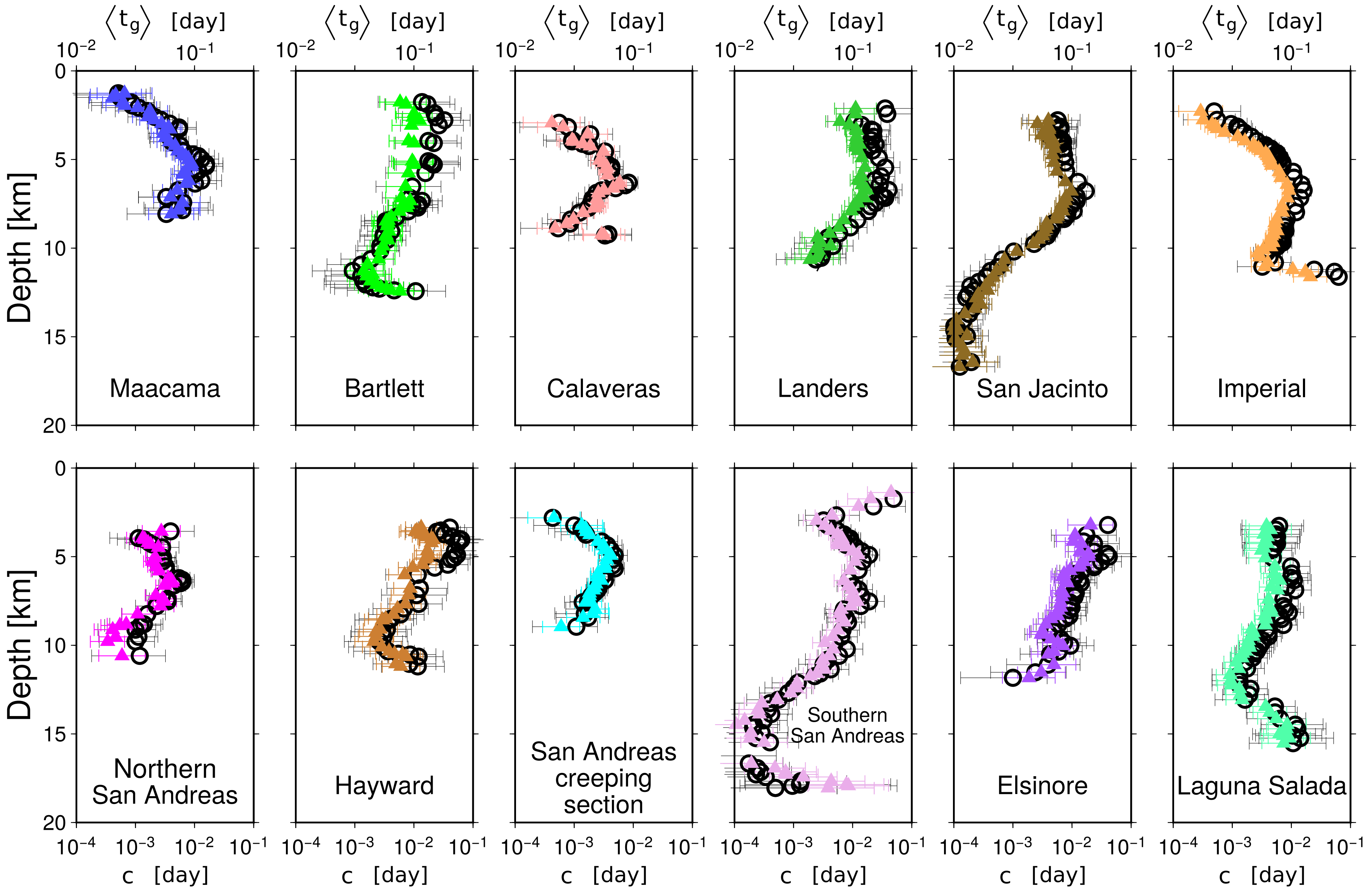


**Supplementary Figure 12:** Comparison between the Bayesian estimates of the $c$-value and the geometric mean $\langle t_g \rangle$ of elapsed times from mainshocks to aftershocks. For each of the 12 sub-vertical strike-slip fault segments studied in Fig. 2 of the main manuscript, the different plots show the dependence on depth of both the $c$-value (black circles) and the $\langle t_g \rangle$-value (colored triangles). Black error bars for $c$-values show the 95% Bayesian credibility regions. Colored error bars for $\langle t_g \rangle$-values show the standard deviation of the sampling distribution.

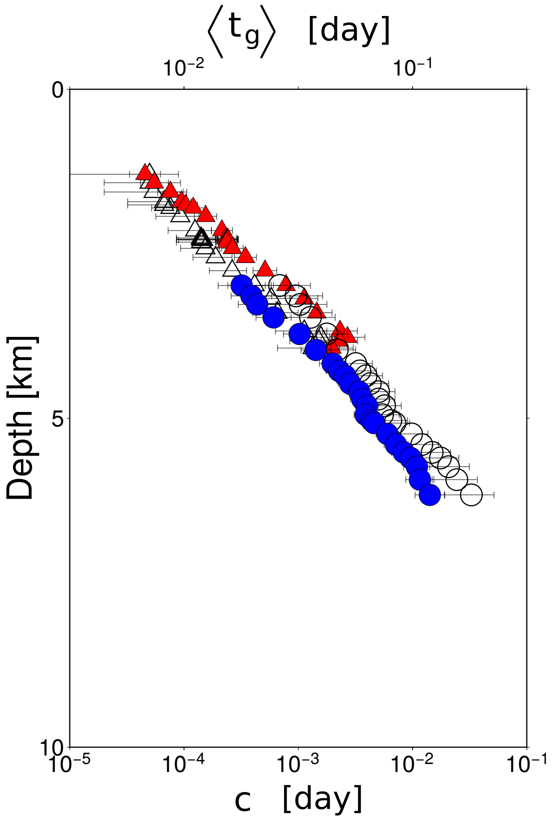


**Supplementary Figure 13:** Comparison between the Bayesian estimates of the *c*-value and the geometric mean $\langle t_g \rangle$ of elapsed times from mainshocks to aftershocks. Red triangles and blue dots show the $\langle t_g \rangle$-values for the Geyser and the Salton Sea geothermal plants, respectively. Colored error bars show the standard deviation of the sampling distribution. Black open triangles (Geyser) and circles (Salton Sea) show the Bayesian estimates of the *c*-value. Black error bars show the 95% Bayesian credibility regions.

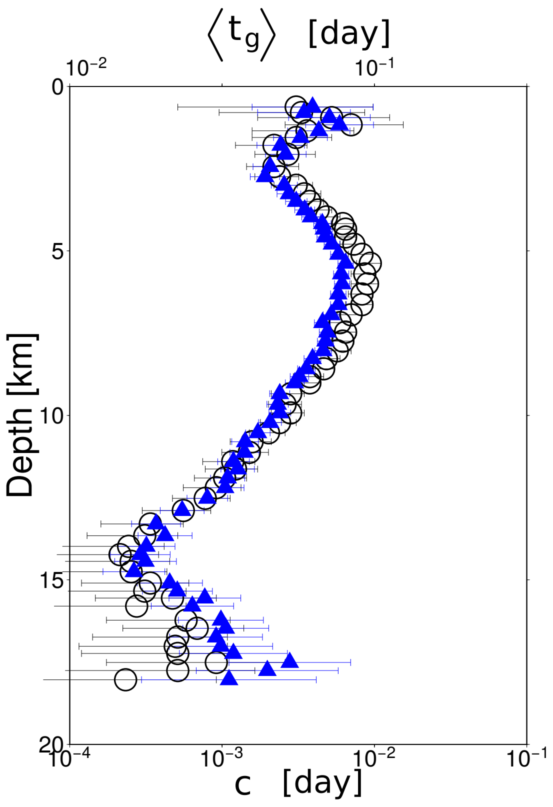


**Supplementary Figure 14:** Comparison between the Bayesian estimates of the *c*-value and the geometric mean $\langle t_g \rangle$ of elapsed times from mainshocks to aftershocks. Blue triangles show the $\langle t_g \rangle$-values considering together all the events occurring along major sub-vertical strike-slip faults in California. Blue error bars show the standard deviation of the sampling distribution. Black circles show the Bayesian estimates of the *c*-value. Black error bars show the 95% Bayesian credibility regions.
